# Incidence of variant hemoglobins in newborns attended by a public health laboratory

**DOI:** 10.1590/S1679-45082018AO4150

**Published:** 2018-05-29

**Authors:** Flávia Mylla de Sousa Reis, Renata Rodrigues de Oliveira Castelo Branco, Amanda Mota Conceição, Letícia Paula Benvindo Trajano, José Felipe Pinheiro do Nascimento Vieira, Pablo Ricardo Barbosa Ferreira, Éverton José Ferreira de Araújo

**Affiliations:** 1 Universidade Federal do Piauí, Teresina, PI, Brazil; 2 Laboratório Central de Saúde Pública Dr. Costa Alvarenga, Teresina, PI, Brazil

**Keywords:** Hemoglobinopathies, Anemia, sickle cell, Neonatal screening, Public health, Mass screening, Infant, newborn, Hemoglobinopatias, Anemia falciforme, Triagem neonatal, Saúde pública, Programas de rastreamento, Recém-nascido

## Abstract

**Objective:**

To evaluate the incidence of variant hemoglobins in different health regions.

**Methods:**

A descriptive, observational, and cross-sectional study with a quantitative approach based on secondary data in the internal records of the neonatal screening service - *Laboratório Central de Saúde Pública do Estado do Piauí* (PI, Brazil). The variables related to sex, ethnicity and positive diagnosis for variant hemoglobins were analyzed, with further population distribution of hemoglobinopathies among the macroregions of the state.

**Results:**

A total of 69,180 samples of newborns were analyzed, and 3,747 were diagnosed as hemoglobinopathies, from February 1^st^, 2014 and December 31^st^, 2015. Sickle cell trait was the most frequent (4.1%), followed by hemoglobinopathy C in 0.9%; homozygous hemoglobin S cases 0.1% stood out and there were no cases of hemoglobinopathy D in the state. It is also worth noting that the highest frequencies of hemoglobin alterations in Piauí were in males (49.8%) and of *parda* skin color (38.5%). The region of Piauí presenting the highest incidence of heteroygous variant hemoglobins was Tabuleiros do Alto Parnaíba and Vale do Sambito, due to importance of the region's population Entre Rios.

**Conclusion:**

Neonatal screening programs are important for screening, orientations regarding health actions and monitoring of families with hemoglobinopathies, in order to reduce morbidity and mortality rates.

## RESUMO

**Objetivo:**

Avaliar a incidência de hemoglobinas variantes em diferentes regiões de saúde.

**Métodos:**

Estudo descritivo, observacional e transversal com abordagem quantitativa baseada em dados secundários presentes nos registros internos do serviço de triagem neonatal do Laboratório Central de Saúde Pública do Estado do Piauí (PI, Brasil). Foram analisadas as variáveis referentes a sexo, etnia e diagnóstico positivo para hemoglobinas variantes, com posterior distribuição populacional das hemoglobinopatias entre as macrorregiões do Estado.

**Resultados:**

Foram analisadas 69.180 amostras de recém-nascidos, e 3.747 diagnosticados com hemoglobinopatias, entre 1° de fevereiro de 2014 e 31 de dezembro de 2015. Constatou-se maior ocorrência do traço falciforme em 4,1% da amostra e da hemoglobinopatia C em 0,9%, com destaque para os casos de hemoglobina S em homozigose (0,1%) e ausência de hemoglobinopatia D no Estado. Destacou-se, ainda, o fato de as maiores frequências de alteração hemoglobínica do Piauí estarem presentes em recém-nascidos do sexo masculino (49,8%) e de etnia parda (38,5%). As regiões do Piauí que apresentaram as maiores incidências de hemoglobinas variantes em heterozigose foram Tabuleiros do Alto Parnaíba e Vale do Sambito, devendo-se ressaltar a relevância populacional da região Entre Rios.

**Conclusão:**

Os programas de triagem neonatal são importantes para rastreamento, orientação de ações de saúde e acompanhamento de famílias acometidas com hemoglobinopatias, a fim de diminuir a morbimortalidade provocada por estas patologias.

## INTRODUCTION

Hemoglobinopathies are clinical conditions that result from structural and functional mutations in the genes that codify globin chains of the hemoglobin (Hb) molecule present in red blood cells.^(^ ^1^ ^)^ Among them, sickle cell disease is the most frequent condition, and the one with the greatest impact due to its high prevalence and severity of its clinical manifestations.^(^ ^2^ ^)^

The first investigation reports of hemoglobinopathies began in 1910, when James Herrick described the presence of elongated and sickle-shaped cells in a blood sample. In a series of children in 1925, Thomas Cooley and Pearl Lee described the clinical thalassemia syndrome with severe anemia, jaundice, and hepatoesplenomegaly. In the following years, the appearance of molecular biology techniques allowed to enhance knowledge about Hb mutations and their corresponding clinical syndromes.^(^ ^3^ ^)^

Several studies showed that the first mutations in Hb molecules appeared on the African continent. In Brazil, the introduction of hemoglobinopathies occurred through the forced immigration of African slaves and the subsequent racial mixture among different population groups, contributing towards the distribution of abnormal genes of globins inherent to the different ethnic groups, and resulting in the overall picture of hemoglobin disorders present in the country today.^(^ ^4^ ^)^

For this reason, the Brazilian population is characterized by several racial origins, diversified degrees of miscegenation, and large-scale genetic heterogeneity. In this way, screenings for hemoglobinopathies should be carried out separately from the ethnic group of the individual under care. However, we emphasize that according to data from the year 2000 census on the classification of skin color or race, black or *“pardo”* (brownish) individuals represent 45% of the Brazilian population.^(^ ^5^ ^–^ ^7^ ^)^

Likewise the overall national scenario, the State of (Piauí, Brazil) displays a history of ethnic miscegenation. The European parental contribution predominates in estimates made for the formation of the population, followed by African, and in a smaller proportion, Indigenous participation.^(^ ^8^ ^)^ Nevertheless, considering that the frequency of hemoglobinopathies in newborns in the State of Piaui is unknown (a fact made evident by the absence of scientific studies published on this theme), as well as the relevance of early laboratory diagnosis of these pathologies, this study aimed to study the incidence of variant hemoglobins in newborns in Piauí.

## OBJECTIVE

To evaluate the incidence of variant hemoglobins in the different regions of the State of Piauí.

## METHODS

This is a descriptive, observational, and cross-sectional study with a quantitative approach, based on secondary data documented in the internal records of the neonatal screening service of the *Laboratório Central de Saúde Pública do Estado do Piauí* (LACEN-PI) *Dr. Costa Alvarenga* [Central Public Health Laboratory of Piauí].

The investigation followed the ethical principles established in Resolution 466/12 of the *Conselho Nacional de Saúde* [National Council on Health], hiding the identity of participants in order to guarantee total anonymity. Data collection began after authorization of the LACEN-PI management and approval of the study by the Research Ethics Committee of the *Universidade Federal do Piauí* (CEP-UFPI), with approval protocol 1.557.038, CAAE# 56004416.5.0000.5214. The investigation waived the application of the Informed Consent Form (ICF), since the study used secondary data from the epidemiological profile and laboratory tests from the neonatal screening sector of LACEN-PI.

Included in the study were all the data related to the period from February 1^st^, 2014, to December 31^st^, 2015, on neonatal screening for hemoglobinopathies produced by LACEN-PI, due to the implementation of the second phase of the screening program know as the Guthrie heel-prick test in the State of Piauí. The variables regarding sex, ethnicity, and positive diagnosis for hemoglobinopathy in Piauí were based on the results of samples from newborns collected on filter paper and analyzed by high-performance liquid chromatography.

**Figure 1:**
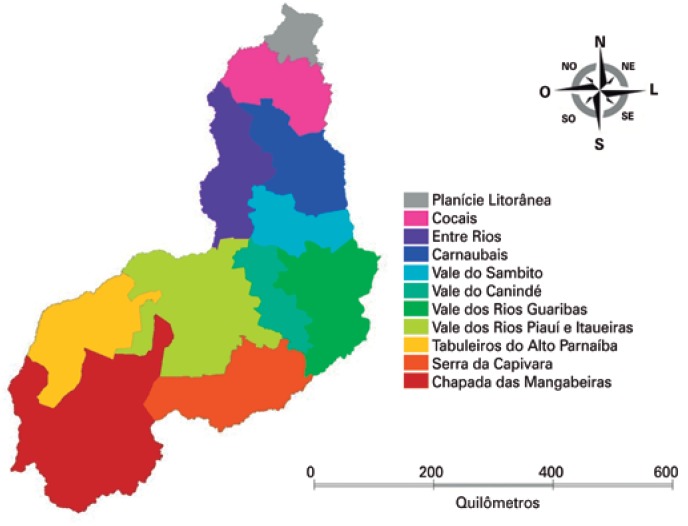
Thematic map of health regions of Piauí

The incidence of the variant hemoglobins, were calculated based on the number of confirmed cases by the number of live newborns for each region of the State. Piauí has 224 cities distributed among 11 health regions and four mesoregions. The North mesoregion encompasses Cocais and the Planície Litorânea; the Central-North mesoregion covers the regions of Entre Rios, Carnaubais, and Vale do Sambito; the Southeast mesoregion includes the Vale do Canindé, Vale do Rio Guaribas, and Serra da Capivara; the Southwest mesoregion includes the regions of the Chapada das Mangabeiras, Tabuleiros do Alto Parnaíba, and Vale dos Rios Piauí e Itaueiras (Figure 1), according to what was established by Supplementary Law #87, dated August 22, 2007,^(^ ^9^ ^)^ which consolidated the Territory Participatory Planning, and determined the said division of the State.

The incidence ranges were used in quartiles, with the objective of geoprocessing the regional incidences. All pieces of information were tabulated on electronic spreadsheets and analyzed as absolute frequency and simple percentage using Microsoft Excel^®^, with further mapping with the aid of the TerraView^®^ program.

## RESULTS

During the period established in the investigation, 69,180 samples were analyzed at LACEN-PI amid 3,747 newborns diagnosed with variant hemoglobins (5.4% of total samples analyzed).

Analyzing the abnormal hemoglobin profiles presented, the one with greatest occurrence was the sickle cell trait (FAS) with 2,848 cases (4.1% of total samples analyzed), followed by the Hb C trait (FAC), present in 613 individuals (0.9%). As to homozygous hemoglobinopathies, sickle cell (FS) anemia stood out with 67 cases (0.1% of total samples analyzed). On the other hand, in Piauí, the occurrence of hemoglobin D disorder (FD) was not noted. Further, there were cases noted of newborns with hemoglobin profiles similar to those of the adult individual, due to late collections. Those who carried non-identifiable Hb were pooled in the category “others.”

Among the newborns diagnosed as having a variant Hb (n=3,747), 1,866 were male (49.8%) and 1,800 female (48.0%); in 56 cases the sex was not informed (1.5%), and 25 had no identification (0.7%). The predominant ethnicity among neonates with abnormal Hb was *“parda”* (38.5%), followed by white (31.%), and 19.6% cases of no ethnicity informed (Table 1).

**Table 1:** Hemoglobin profiles, sex and ethnicity of newborns diagnosed with variant hemoglobins

Parameters		n
Hemoglobin profile		
	Hemoglobin S trait	2,848
	Hemoglobin C trait	613
	Hemoglobin D trait	68
	Hemoglobin E trait	11
	Hemoglobin Barts	2
	Sickle cell disease	67
	Hemoglobin C disease	5
	Hemoglobin S and C disease	19
	Hemoglobin D disease	0
	Hemoglobin E disease	0
	Others	114
Sex		
	Male	1,866
	Female	1,800
	Not informed	56
	With no identification	25
Ethnicity		
	Black	199
	*Parda*	1,443
	Indigenous	18
	White	1,187
	Yellow	142
	Not informed	733
	With no identification	25

**Figure 2:**
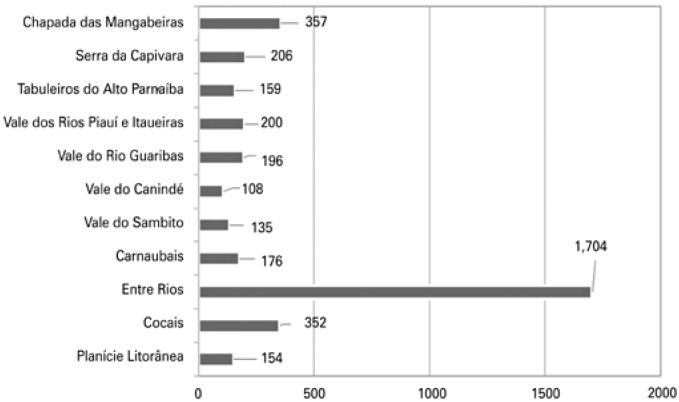
Births with variant hemoglobins per health regions of Piauí

Figure 2 shows the distribution, by health regions, of total number of newborns diagnosed with variant Hb in Piauí. This clinical condition was more often observed in the regions of Entre Rios (45.5%), Chapada das Mangabeiras (9.5%), and Cocais (9.4%).

**Figure 3:**
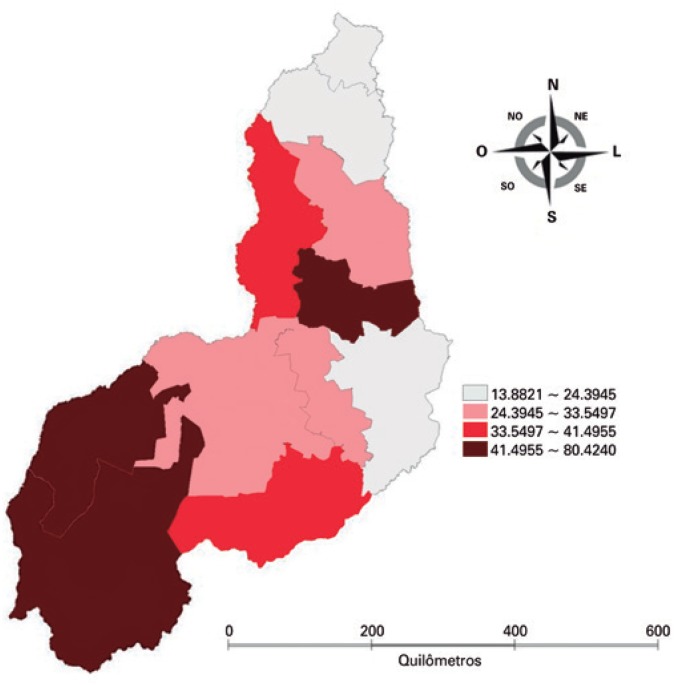
Thematic map of incidence (per 1,000 liveborns) of hemoglobin profile sickle cell trait distributed per health regions of Piauí

**Figure 4:**
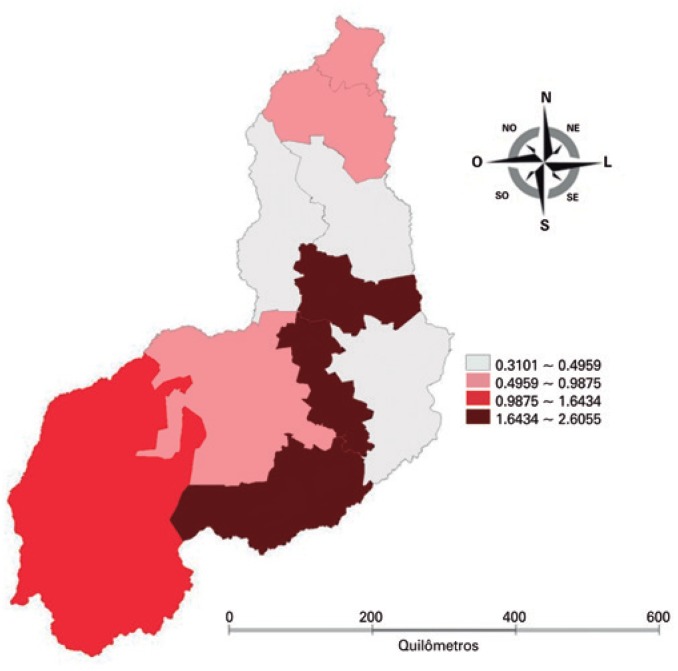
Thematic map of incidence (per 1,000 liveborns) of hemoglobin profile of sickle cell disease distributed per health regions of Piauí

Among the variants hemoglobins, there was a greater incidence of the S variant as compared to other hemoglobinopathies. What about sickle cell disease, higher incidence in the region of Tabuleiros do Alto Parnaíba, with 80.4 per 1,000 liveborns, followed by Chapada das Mangabeiras and Vale do Sambito, with 44.1 and 41.5, respectively, per 1,000 liveborns. The FS profile predominated in Serra da Capivara, Vale do Canindé, and Vale do Sambito with 2.6, 1.8, and 1.6 per 1,000 liveborns, respectively (Figures 3 and 4).

## DISCUSSION

Effective neonatal screening enables early diagnosis for identification of variant Hb carriers and their inclusion into specialized care programs, aiming to reduce morbidity and mortality related to hemoglobinopathies.^(^ ^10^ ^)^

In this scenario, it is initially important to point out the presence of patients with “not informed” and “no identification” profiles regarding ethnicity and sex. Patients classified as having “no identification” did not present with any data such as name of mother, ethnicity or sex, which could be related to errors in the information management system of LACEN-PI, or in the registration of the neonatal screening program.

Whereas the patients with the “not informed” profiles did not present data on ethnicity and/or sex, as a result of not complying with data gathering during sample collection for the Guthrie heel-prick test. This is a relevant fact, since it hinders analysis of the information derived from this healthcare service, since hemoglobinopathies show a direct relation with ethnic aspects.

The Piauí regions with lowest development indices presented with the largest quantities of incomplete registration cards regarding information on ethnicity, which suggests a lack of understanding as to the importance of this information for screening hemoglobinopathies. Availability of information based on valid and reliable data is an essential condition for the objective analysis of the clinical situation, as well as for evidence based decision-making and programming public health actions. Therefore, it ratifies the importance of healthcare professionals qualified for transmitting and conducting the correct acquisition of information during the completion of the registration cards upon collection, suggesting the need for continuing education of staff involved in neonatal screening in Piauí.^(^ ^11^ ^)^

The State of Piauí presented the highest frequency of newborns with heterozygosis for Hb S (FAS), which is important clinical information for identifying families at risk of generating children with sickle cell disease, followed by heterozygosis for Hb C (FAC) and Hb D (FAD), and homozygosis for Hb S (FS).

This prevalence of the FAS, FAC, and FS among the neonates studied was considerable and consistent with the results found in studies carried out both in other Brazilian States and overseas. This demonstrates the relevance of maintenance and amplification of screening and diagnosis programs focused on reducing morbidity and mortality of patients, especially those with sickle cell disease, as well as enabling implementation of effective genetic counseling programs.^(^ ^12^ ^)^

Historically, phenotypes of hemoglobinopathies S and C originated from Africa, where the highest prevalence persists. In Equatorial Africa, for example, 40% of the population carries the Hb S gene, and sickle cell disease affects 2 to 3% of population. This phenotypical occurrence in the Americas results from immigration processes, and in Brazil, it is more frequent in the Southeast and Northeast Regions.^(^ ^5^ ^,^ ^13^ ^,^ ^14^ ^)^

The study done by Lobo et al.,^(^ ^2^ ^)^ carried out in the State of Rio de Janeiro with 99,260 screened newborns, demonstrated that 3.96% of neonates presented FAS and 588 (0.59%), FAC; 0.06% of patients had a FS profile and only 0.01% were FC. The research carried out by Diniz et al.,^(^ ^15^ ^)^ on the other hand, demonstrated that in the Federal District, from 2004 to 2006, a total of 116,271 neonates were screened, and 3.2% of them presented the sickle cell trait, and 0.09% carried the sickle cell disease, similar to what was observed in this study.

Relative to the international scenario, Piauí shows a high incidence of the sickle cell trait, when compared to Mediterranean countries, a fact justified by the late immigration to the European countries and the predominance of the Caucasian population. The study performed by Cela et al.,^(^ ^16^ ^)^ in Spain, for example, noted a reduced occurrence of hemoglobinopathies, with approximately 1.5 cases per 100 thousand individuals. On the other hand, the study by Costa et al.,^(^ ^17^ ^)^ conducted at *Maternidade Alfredo da Costa*, in Portugal, demonstrated, based on the analysis of umbilical cord blood samples from 400 newborns, only 1.5% of cases of heterozygosis for Hb S, four of which were of African origin. Moreover, there was no case of sickle cell disease, corroborating literature data of low incidence of this disease in Europe, a continent that shows the highest presence of thalassemia.^(^ ^18^ ^)^

Conversely, in comparison with the Asian continent, for example, in India, where the population is mixed, recent research using the high-performance liquid chromatography methodology analyzed 25,297 samples and determined the predominance of the sickle cell trait among the Indian population (33.03%) − as is observed in this study.^(^ ^19^ ^)^ Taking this into consideration, studies on the incidence of variant Hb are fundamental to check occurrences and potential risk factors, besides providing an overview of the screening programs, and the risks to which the population of each region is posed to, especially to homozygous forms, in which early clinical intervention favors improvement in the patient's quality of life.^(^ ^6^ ^,^ ^20^ ^)^

Albeit knowing that the gene that codifies the globin chains is not linked to sex, male newborns (49.8%) accounted for most cases of variant Hb in Piauí, similar, in this aspect, to the research by Pimentel et al.^(^ ^21^ ^)^ Researchers observed that in Bahia, between 2007 and 2009, a total of 581,060 newborns were screened, and of these, 966 (0.16%) presented with the sickle cell profile; 374 were diagnosed in 2009; 311 in 2008; and 281 in 2007. Among the newborns diagnosed, the majority (50.1%) was male and presented with the sickle cell disease in homozygosis (FS).^(^ ^21^ ^,^ ^22^ ^)^

Despite the fact that Piauí has a strong presence of black population, including with maintenance of *quilombola* communities (which reflects ethnic similarities with the Bahia State population), it was possible to perceive that, unlike the study by Amorim et al.,^(^ ^21^ ^)^ the present investigation indicates a majority of individuals with Hb S in heterozygosis (FAS). Most patients with a FAS profile are in conformity with the results of Soares et al.,^(^ ^23^ ^)^ who pointed out a higher occurrence of the sickle cell trait in the investigation of variant hemoglobins in 1,000 samples obtained at the *Centro de Hematologia e Hemoterapia do Estado do Piauí* (HEMOPI).^(^ ^20^ ^,^ ^24^ ^)^

In the State of Piauí, the mesoregions North, Center-North, Southeast, and Southwest, showed the contribution of European, African, and Indigenous ancestry. This datum reinforces the results obtained, in which the frequency of Hb S in all Piauí regions is reinforced, a fact justified by the participation of African ancestry in the State as a whole. Further, it was noted that in the 11 Piauí regions, there was a greater frequency of *parda* and white ethnicities, which reinforces the accentuated racial miscegenation of the population.^(^ ^8^ ^)^ The regions with the highest incidences of sickle cell trait and of sickle cell disease should be considered priority for the adoption of health promotion measures directed at sickle cell patients.

The FS form is the most well known hemoglobinopathy, and it is considered a public health issue of great epidemiological importance. It is associated with the high morbidity and mortality in childhood, due to hindered oxygen transport, vaso-occlusions, bacterial sepsis, splenic sequestration crisis, and acute thoracic syndrome. Thus, greater effectiveness of the National Neonatal Screening Program is crucial, with the implementation of preventive measures and ongoing follow-up of affected patients, thus assuring a better quality of life.^(^ ^5^ ^,^ ^25^ ^)^

As to the incidence of FAC, Tabuleiros do Alto Parnaíba, Chapada das Mangabeiras, and Vale do Sambito had higher incidence values with 11.2, 10.2, and 8.2 per 1,000 liveborns, respectively. These patients were asymptomatic. Whereas in the cases of FC, in which the patients presented with variable hemolytic anemia, the incidence in Chapada das Mangabeiras, Planície Litorânea, and Entre Rios was 0.5, 0.1, and 0.03 per 1,000 liveborns, respectively.

Sickle cell trait and FAE cases corresponded to rare heterozygous hemoglobinopathies with an asymptomatic condition, generally tracked by genetic studies in the population.^(^ ^26^ ^)^ The highest incidence of the FAD profile was noted in Tabuleiros do Alto Parnaíba, with 2.5 per 1,000 liveborns, followed by Carnaubais and Vale do Sambito, with 1.3 and 1.2 per 1,000 liveborns, respectively. The incidence of FAE, on the other hand, was highest in Vale do Sambito, followed by Tabuleiros do Alto Parnaíba, Vale do Rio Piauí, and Itaueiras, showing 1.2, 0.6, and 0.4 per 1,000 liveborns, respectively.

With the data obtained, greater indices of variant Hb were noted in Piauí in the regions of Tabuleiros do Alto Parnaíba, Serra da Capivara, Chapada das Mangabeiras, Vale do Canindé, and Vale do Sambito, emphasizing the region of Entre Rios due to its high population density and the large absolute number of cases, especially FAS and FAC, likely resulting from the city of Teresina, the most populous of the State, with more than 800 thousand inhabitants.^(^ ^27^ ^)^

## CONCLUSION

This present study showed a predominance of *“parda”* skin color male in neonates with variant hemoglobins, especially greater frequency of hemoglobin S, both in heterozygosis and in homozygosis. There were no cases of hemoglobinopathies D and E t in Piauí, which highlights the importance of the high incidence of sickle cell disease in the State. The most populous cities of the regions with the highest indices of hemoglobin variants should be prioritized in healthcare actions; especially in the regions of Tabuleiros do Alto Parnaíba and Serra da Capivara, frontier regions with the States of Maranhão and Bahia, largely inhabited by blacks and who presented with the highest incidences of sickle cell disease and sickle cell neonates, respectively. Additionally, we point out the Entre Rios region, since it hosts the largest absolute number of cases with the presence of some variant hemoglobin, a region in which the capital of Piauí, Teresina, is located.
